# Analyzing Ash Leaf-Colonizing Fungal Communities for Their Biological Control of *Hymenoscyphus fraxineus*

**DOI:** 10.3389/fmicb.2020.590944

**Published:** 2020-10-22

**Authors:** Regina Becker, Kristina Ulrich, Undine Behrendt, Michael Kube, Andreas Ulrich

**Affiliations:** ^1^Microbial Biogeochemistry, Research Area Landscape Functioning, Leibniz Centre for Agricultural Landscape Research (ZALF), Müncheberg, Germany; ^2^Institute of Forest Genetics, Johann Heinrich von Thünen Institute, Waldsieversdorf, Germany; ^3^Integrative Infection Biology Crops-Livestock, University of Hohenheim, Stuttgart, Germany

**Keywords:** ash dieback, antagonism, *Fraxinus excelsior*, mycobiome, cocultivation, phyllosphere

## Abstract

The invasive ascomycete *Hymenoscyphus fraxineus* has been threatening *Fraxinus excelsior* populations throughout Europe for over two decades. Since the infection and first colonization by the pathogen occurs in leaves, leaf-colonizing microorganisms have been discussed as a barrier and as possible biocontrol agents against the disease. To identify fungal groups with health-supporting potential, we compared the fungal microbiota of compound leaves from susceptible and tolerant ash trees in four ash stands with high *H. fraxineus* exposure. The fungal communities were analyzed both culture-independently by ITS2 amplicon sequencing and by the taxonomic classification of 1,704 isolates using matrix-assisted laser desorption/ionization time of flight mass spectrometry (MALDI-TOF MS) or sequencing of the entire ITS region. The fungal community structure did not show significant differences depending on the health status. However, for several OTUs and a MALDI group, a significantly higher abundance was found in tolerant ash trees. Thus, the yeast *Papiliotrema flavescens* was significantly increased and accounted for 12.3% of the mycobiome of tolerant ashes (OTU0003), and it had also a distinctly higher abundance among the isolates. The filamentous ascomycete *Sarocladium strictum* was increased 24-fold among the isolates of tolerant trees, but its abundance was comparably low. An *in vitro* screening for the growth inhibition of the pathogen *via* cocultivation resulted in 28 yeast-like isolates and 79 filamentous fungi with antagonistic activity. A statistical cocultivation test on two *H. fraxineus* strains confirmed six of the yeast-like isolates that suppressed *H. fraxineus* significantly, from 39–50%, two of them through a fungicidal effect. The highest inhibition rates among the yeasts were found for three isolates belonging to *Aureobasidium pullulans* and *P. flavescens*. The cocultivation test of the filamentous isolates revealed higher effects compared to the yeasts. Four isolates showed significant inhibition of both *H. fraxineus* strains with a rate of 72–100%, and five further isolates inhibited only one *H. fraxineus* strain significantly. The most effective isolates were members of the genus *Cladosporium*. During the next step, *in planta* tests will be necessary to verify the efficacy of the antagonistic isolates and to assess their suitability as biocontrol agents.

## Introduction

In recent decades, ash dieback has spread from north-eastern Poland to all over Europe, and it threatens the future of the common ash (*Fraxinus excelsior*) because of its associated severe damage and high mortality rates (Kowalski and Holdenrieder, 2009; Timmermann et al., 2011; McKinney et al., 2014; Skovsgaard et al., 2017). This disease is caused by the invasive ascomycete *Hymenoscyphus fraxineus* (Kowalski, 2006; Queloz et al., 2011; Baral et al., 2014). The fungus was introduced from East Asia, where it lives in asymptomatic association with the Manchurian ash (*Fraxinus mandshurica*) (Zhao et al., 2013; Gross et al., 2014; Han et al., 2014; Cleary et al., 2016). *H. fraxineus* infections come from airborne ascospores that germinate on leaves and penetrate the cuticula and the epidermis. Starting from the infected leaves, the pathogen is able to colonize the petioles and subsequently the twigs and shoots. This leads to blockages of the xylem vessels as well as lesions of the cambium and finally the crown starts dying back (Gross et al., 2014). Infection and colonization by the pathogen can be prevented or reduced by genetically mediated defense mechanisms. Thus, several ash species, such as *F*. *mandshurica* and *Fraxinus ornus*, the native flowering ash, tolerate this pathogen based on weak susceptibility (Gross et al., 2014; Kirisits and Schwanda, 2015; Drenkhan et al., 2017). A hereditary resilience seems to also be present within the common ash, which is reflected in the occurrence of individual healthy-looking trees in infested stands (Stener, 2013; Lobo et al., 2014; Wohlmuth et al., 2018). Resistance is considered as a polygenic trait, the specific function of which is still poorly understood (Wohlmuth et al., 2018; Stocks et al., 2019). However, it is assumed that the resilience of the host is significantly influenced by its microbiome (Berg et al., 2017; Compant et al., 2019; Liu et al., 2020). Bacteria and fungi that colonize the surface and the endosphere of the plant can suppress invaders directly by resource competition, parasitism and antibiosis or indirectly by activating the plant’s own defense system (Latz et al., 2018; Fadiji and Babalola, 2020). In addition, some endophytic microorganisms are able to promote plant growth and development, resulting in an increased resilience in response to stress and pathogens (Glick, 2012; Hossain et al., 2017). These different mechanisms, which can occur simultaneously, are activated in interaction with the plant’s metabolism and the environmental conditions (Berg and Smalla, 2009; Berg et al., 2015).

To assess the role of fungal endophytes in ash dieback tolerance, the structure of leaf- and twig-associated fungal communities of tolerant and diseased ashes was investigated over a series of studies (Cross et al., 2017; Hanackova et al., 2017a; Schlegel et al., 2018; Kosawang et al., 2019). Different approaches showed significant variations in the community structure depending on the plant location, season and plant genotype. In particular, the composition of twig-associated fungal communities differed significantly between ash species, i.e., *Fraxinus angustifolia*, *Fraxinus latifolia*, *F*. *mandshurica*, and *F. ornus* but also between genotypes of *F. excelsior*. However, a direct and distinctive relation to ash dieback susceptibility could not be detected (Kosawang et al., 2019; Griffiths et al., 2020). Griffiths et al. (2020) observed that higher *H. fraxineus* infection levels were associated with higher fungal diversity and the presence of specific fungal genera.

The complex interactions between the resistance, plant genotype and associated microorganisms make it challenging to identify specific microbial groups with disease-suppressing potential. It is assumed that health-supporting taxa should primarily be present in tolerant hosts (Schulz et al., 2019). However, only a few species have been identified so far, which are clearly associated with *H. fraxineus* tolerance. Cleary et al. (2016) analyzed the phyllosphere fungal communities of the dieback-tolerant ash *F*. *mandshurica* and identified *Mycosphaerella* as the dominant fungal genus in these trees. *Mycosphaerella* was found in all the *F*. *mandshurica* samples collected in eastern Russia and was limited to this ash species. A study on the leaf petioles of common ashes showed increased frequencies of *Aureobasidium pullulans* in healthy trees, while in diseased trees, *Alternaria* and *Cytospora* species were also frequently represented (Davydenko et al., 2013). Other investigations focused on the *in vitro* testing of ash leaf endophytes for antagonism against *H. fraxineus*. A row of fungal isolates obtained from tolerant ash species as well as *F. excelsior* indicated antagonistic activity in cocultivation with the pathogen (Hanackova et al., 2017a; Kosawang et al., 2018; Halecker et al., 2020). However, cocultivation experiments also showed that both partners, *H. fraxineus* and the endophytic isolate, are often inhibited by mutual antagonisms (Schulz et al., 2015; Halecker et al., 2020). Therefore, comprehensive tests are required to identify effective antagonistic candidates whose colonization and activities are not or only weakly suppressed by *H. fraxineus.*

In this study, we combined a high-throughput sequencing approach and cultivation methods to analyze the fungal communities of susceptible and tolerant common ashes. The comparative analyses were aimed at detecting the fungal groups that are specific to tolerant trees and might therefore be involved in disease suppression. In parallel, the isolates were subjected to an extensive screening for *H. fraxineus* growth inhibition. Based on the primary screening, the best isolates were evaluated in a statistical cocultivation test for their antagonism against the pathogen.

## Materials and Methods

### Study Site and Sampling

Compound ash leaf material was obtained by sampling of ash trees from four forest districts in Northeast Germany in July of 2017. Observations taken during long-term monitoring projects revealed a very low occurrence of single trees with low susceptibility in stands that were severely affected by *H. fraxineus* (Sündermann and Jütte, 2014). In each of the districts (A–D), four pairs of adjacent trees consisting of an affected (K) and a tolerant (P) tree were chosen for sampling. Details of the sampling campaign and sample preparation are described in Ulrich et al. (2020). Accordingly, the same leaf samples were used in this study to analyze the fungal community.

### Amplicon Sequencing and Analysis of the ITS2 Region

For high comparability among analyses, the total DNA used to investigate of the bacterial community (Ulrich et al., 2020) was also applied to study the fungal community. For this purpose, the ITS2 region was chosen because it has a lower length variability compared to the ITS1 region combined with higher genetic diversity. It also reduces the pitfalls caused by introns in the flanking rDNA of the ITS1 region of some species (Tedersoo et al., 2015). The ITS2 region was amplified with the primers fITS7 (5′-GTGARTCATCGAATCTTTG-3′) (Ihrmark et al., 2012) and ITS4 (5′-TCCTCCGCTTATTGATATGC-3′) (White et al., 1990), preventing the amplification of the host plant DNA. PCR amplification was conducted as described by Ihrmark et al. (2012). The primers were extended with a heterogeneity spacer and a barcode sequence. The amplicons were checked with gel electrophoresis, purified with an MSB Spin PCRapace kit (Invitek, Essen, Germany) and mixed to equimolar DNA concentrations. The library preparation and Illumina MiSeq 300-bp paired-end sequencing were performed at LGC Genomics Berlin.

The raw sequence data were analyzed with Mothur v. 1.39.1 (Schloss et al., 2009; RRID:SCR_011947) in accordance with the MiSeq SOP (Kozich et al., 2013). In addition, the ITS2 region was extracted with ITSx 1.1.2 (Bengtsson-Palme et al., 2013) by removing the flanking 5.8S and 28S rRNA gene fragments. Chimeras were removed using the Uchime algorithm. For phylogenetic identification, the sequences were compared to the dynamic UNITE database (version 8.2) using a confidence threshold of 80%. The operational taxonomic units (OTUs) were generated with the VSEARCH algorithm without using a multiple sequence alignment (Edgar, 2010) at a similarity of 97%. Singletons were discarded as suggested in the UPARSE pipeline (Edgar, 2013). The phylogenetic assignment of the OTUs was checked manually using BLAST searches of the UNITE database as well as the NCBI taxonomy browser. All the samples were subsampled to the minimum number of sequences among all the samples. The paired sequence reads were deposited in the public Sequence Read Archive (SRA, RRID:SCR_004891) repository under the accession number PRJNA611938.

Statistical analyses were performed using the phyloseq, vegan and ape packages in R 3.6.0, as well as MicrobiomeAnalyst (Chong et al., 2020; RRID:SCR_015022). The alpha diversity indices Chao 1, Shannon, and Simpson were calculated. Significant differences between the fungal communities were tested using an Analysis of Similarity (ANOSIM). The phylogenetic composition of the ash microbiome was visualized with a Krona chart (Ondov et al., 2011). The differential abundance at different taxonomic levels was analyzed with the metagenomeSeq tool using a false discovery rate (FDR) for multiple test correction (Paulson et al., 2013). An indicator species analysis was performed with PC-Ord v. 7.02 (McCune and Mefford, 2015) by applying the procedure by Dufrêne and Legendre (1997).

### Cultivation of Fungal Epi/Endophytes

Ground material of compound ash leaves were serially diluted in 0.8% NaCl, plated on PDA (Merck, Darmstadt) and incubated for seven to ten days at room temperature. To determine the population densities, colony forming units (CFU) were counted and expressed as CFU per gram of fresh weight. Approximately 50 isolates per sample were randomly selected for analysis. From the filamentous fungi, the mycelium was subcultivated on PDA and pure cultures were scraped from the Petri dishes using a sterile scalpel and transferred to agar slants (PDA) for storage and further processing. Yeast-like fungi were purified by streak plate method and stored as cryo-cultures (−80°C) in nutrient broth containing 40% glycerol until analysis.

### Dereplication and Classification of Fungal Isolates

Matrix-assisted laser desorption/ionization time of flight mass spectrometry (MALDI-TOF MS) was applied as a reliable method for the dereplication and classification of yeasts in environmental analyses (Chudejova et al., 2017; Huschek and Witzel, 2019). Prior to this measurement, the yeast-like isolates were freshly inoculated on CASO agar (Fluka, Buchs, Switzerland) and cultivated for 24 h at 25°C. Mass spectra were obtained based on the whole-cell measurement protocol as described by Ulrich et al. (2020). The analysis was performed on a microflex^TM^ LT/SH MALDI-TOF mass spectrometer (Bruker Daltonics, Bremen, Germany) using Flex Control 3.4 software. Isolates showing spectra with a score value >2.3 (highly probable species identification) were considered as identified by the Bruker database. Isolates with a lower score were compared with each other and grouped on the basis of a score value >2.3.

Representative strains of the unidentified groups were taxonomically classified by sequencing of the complete fungal ITS rRNA region. Total DNA was extracted from single colonies by resuspending in 20 μl of 25 mM NaOH/0.25% SDS followed by incubation for 15 min at 95°C. The amplification was performed using the universal primer pair ITS1F (Gardes and Bruns, 1993) and ITS4 (White et al., 1990). PCRs were performed using PCR Master Mix 2× (Thermo Fisher Scientific, Darmstadt, Germany) containing 0.2 μM of each primer. The thermal cycling conditions were as follows: an initial denaturation step of 95°C for 5 min; 10 cycles of 95°C for 30 s, 60°C for 30 s with a touchdown of −1°C by cycle and 72°C for 1 min, 30 cycles of 95°C for 30 s, 50°C for 30 s and 72°C for 1 min. The PCR products were checked on a 1% agarose gel and the fragments of approximately 600–700 bp were sequenced using the primer ITS1F. The fungal isolates were taxonomically assigned using the UNITE fungal ITS database version 8.0 (Kõljalg et al., 2013) and the species names were approved by the NCBI taxonomy browser. After supplementing the MALDI BiotyperTM database with the spectra of the reference strains, a reliable taxonomic identification of all the fungal isolates was achieved. All the MALDI groups were assigned at the species level.

For the classification of filamentous fungi, small pieces of mycelia (ca. 0.5 cm^2^) were scraped from agar cultures using a sterile scalpel. To extract the DNA, a modified CTAB protocol was used according to Doyle (1990). In brief, for the first lysis step, the mycelium was frozen at −80°C in 2 ml tubes containing 8–10 ceramic beads (1.4 mm; Omni International, Kennesaw, United States) and subsequently ground for 25 s with a speed of 7.45 m/s using the Bead Ruptor 24 (Omni International). After the addition of 750 μL of CTAB buffer and sample incubation of the samples for 30 min at 65°C, the procedure followed the regular protocol. Filamentous fungal isolates were identified by sequencing the entire ITS region of ribosomal DNA as described above and assigned at the species level as well.

A community analysis of culturable fungi was performed by combining all the isolates classified by either MALDI-TOF MS or direct sequencing. The B2P sample was removed from the analysis as an outlier due to the small number of only seven isolates. For the statistical analysis, the number of isolates per sample was normalized using the CSS method (Paulson et al., 2013). Significant differences in the community composition between tolerant and susceptible trees were tested at all taxonomic levels using the MetagenomeSeq tool (MicrobiomeAnalyst, *P*_*FDR*_ < 0.05).

### *In vitro* Screening and Statistical Test for Antagonistic Activity Against *H. fraxineus*

All the fungal isolates were screened for antagonistic activity against *H. fraxineus* P3. To simulate the conditions of their natural habitat, cultivation was performed on PDA medium enriched with ash shoot extract (30 g ash leaves/L PDA) at 22°C (Hanackova et al., 2017a). Due to its slow growth, *H. fraxineus* P3 was incubated for 5 to 6 days before the isolates for testing were struck out. Depending on the growth type of the isolates (yeasts or filamentous fungi), two different approaches were used:

The antagonistic potential of yeast-like fungi was estimated using an assay according to Pane and Zaccardelli (2015). An agar plug (ø 5 mm) containing fresh *H. fraxineus* mycelium was placed in the center of the Petri dish (ø 8 cm), whereas four different yeast counterparts were spread around the four edges of the plate. Plates, solely carrying *H. fraxineus* served as control. The colony radius (r) of *H. fraxineus* was measured after seven and 14 days until the control reached the edge of the plate. The inhibitory effect was estimated using the formula percent growth inhibition = 100 × [(*r*_*control*_ – *r*_*cocultivation*_)/*r*_*control*_]. Isolates with inhibition rates >30% were chosen for a subsequent statistical test including three replicates and two *H. fraxineus* strains (P3, HF23). In this approach, only one isolate per plate was spread out along the edges of the plate, and the inhibition rates were calculated by measuring the diameter of the *H. fraxineus* mycelium.

The filamentous fungi were tested in dual cultures with agar plugs (ø 5 mm) of the fungal isolates and *H. fraxineus* mycelium at a distance of 4 cm. The test included a self-inhibition control by pairing two *H. fraxineus* colonies of the same strain and a negative control by pairing *H. fraxineus* with an agar plug without mycelium. Similar to Hanackova et al. (2017a), the growth of *H. fraxineus* was measured as the colony radius on a connective line between the two colonies as well as on a line measuring 45° up and down from this line after seven and 14 days of dual culture. The inhibition of *H. fraxineus* was estimated after averaging the three measured values and using the formula mentioned above. Isolates that indicated higher inhibition rates compared to the self-inhibition were evaluated again in a statistical test, as outlined above.

In addition, the statistical tests on the yeasts and filamentous fungi were ultimately used to evaluate the vitality of the residual *H. fraxineus* mycelium after confrontation with the fungal isolates. For this purpose, agar plugs with the remaining *H. fraxineus* mycelium (P3, HF23) were picked from the three replicates and incubated separately on PDA with ash shoot extract. The growth of the mycelium was assessed after 2 weeks in comparison to the untreated control.

To verify the pathogenicity of the *H. fraxineus* isolates P3 and HF23, 2-year-old ash seedlings were inoculated with either isolate using an approach modified according to Przybyl (2003). The typical symptoms of ash dieback (Gross et al., 2014) were monitored over the course of 8 weeks, and the virulence of both isolates was confirmed.

## Results

### Fungal Community Structure of Susceptible and Tolerant Ashes

#### ITS2-Based Community Profiling

The mycobiomes of 16 tolerant and 16 susceptible trees were studied by amplicon sequencing of the ITS2 rRNA region. Although only visibly healthy leaves and petioles were sampled from both the tolerant and the diseased trees, an extremely high proportion of *H. fraxineus* (86%) was found in the fungal community of one sample (C2K). Due to this contamination, this sample was excluded from further analysis. All the other samples contained a low proportion of the pathogen *H. fraxineus* with an average of 0.03% of the fungal community. In total, the sequences could be assigned to 1012 operational taxonomic units (OTUs) with an average of 210 OTUs per sample. For the statistical analyses, the sequences were rarefied to 10,707 reads per sample.

**FIGURE 1:**
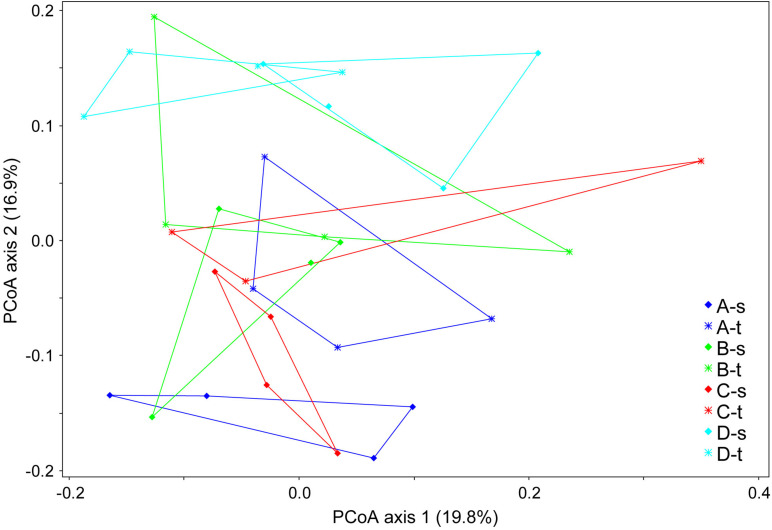
Community clustering of the mycobiome of tolerant **(t)** and susceptible **(s)** ashes from the four forest districts **(A–D)**. A principle coordinate analysis (PCoA) was applied based on a Bray-Curtis distance matrix.

The comparison of fungal communities from tolerant and susceptible trees showed no significant differences in the genetic diversity (e.g., with a mean Shannon index of 2.72 ± 0.23 vs. 2.65 ± 0.44). An analysis of the composition of the mycobiome revealed small but significant differences in grouping by sample location (forest districts) and health status (ANOSIM *R* = 0.1898, *P* < 0.001). Figure 1 displays the differences in the fungal community structure. The second axis indicated some deviations between tolerant and susceptible ashes for forest districts A and C. However, the pairwise comparisons of the health status did not show significant differences for any of the districts.

**FIGURE 2:**
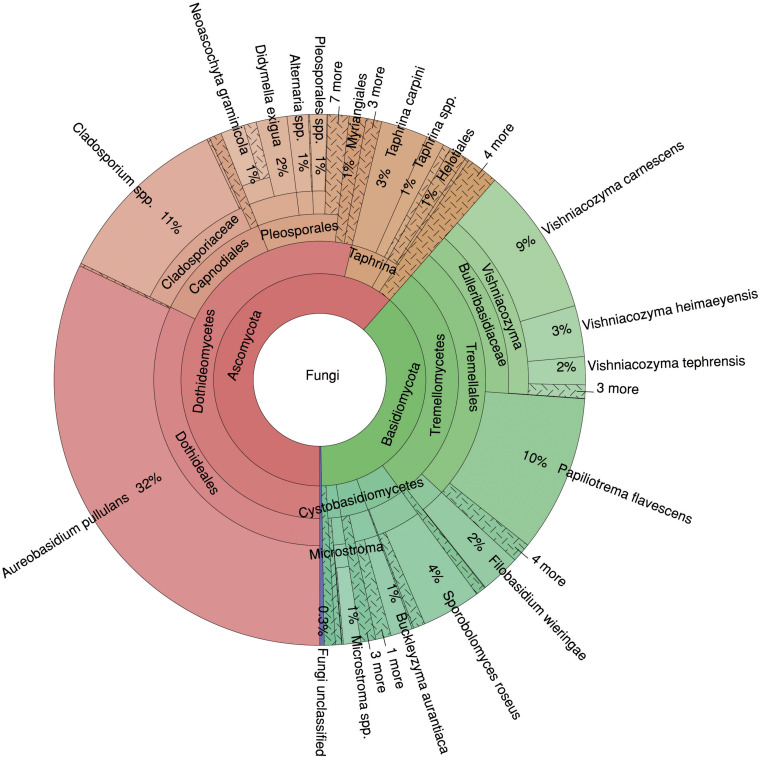
Relative proportions of taxa in the mycobiome of *Fraxinus excelsior*. The Krona diagram shows six taxonomic levels from phylum to species.

In total, 61% of the sequences were assigned to the phylum *Ascomycota* and 38% to the phylum *Basidiomycota*, and only 0.46% of the fungal sequences could not be classified (Figure 2). At the class level, *Dothideomycetes* dominated the fungal community with 54%, followed by *Tremellomycetes* with 28%. At the species level, *A. pullulans* showed the highest abundance with 32%, followed by *Cladosporium* spp. (11%), *Papiliotrema flavescens* (10%) and *Vishniacozyma carnescens* (9%). Thus, four species represented more than 60% of the fungal community.

**FIGURE 3:**
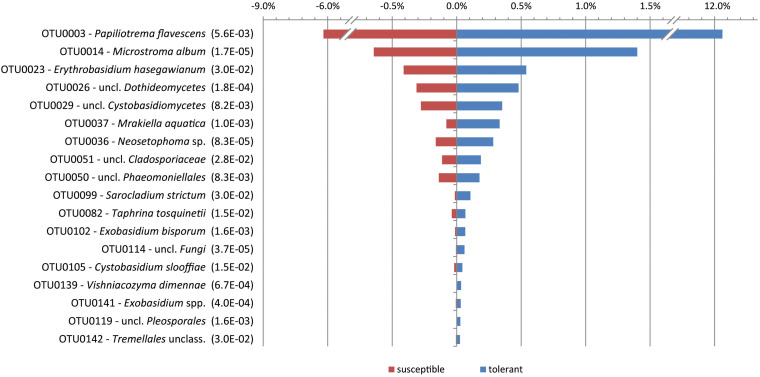
Significantly increased OTUs in tolerant ashes. The OTUs are sorted according to their relative abundance (>0.02), and the level of significance (FDR) is given in brackets.

When comparing the mycobiome at the species level, only the yeast *P. flavescens* was significantly increased in tolerant trees (approximately 2-fold). At the OTU level, the differential abundance analysis revealed 18 OTUs with a proportion of more than 0.02% that were significantly increased in tolerant trees (Figure 3). Remarkably, a group identified as *P. flavescens* (OTU0003) accounted for 12.3% of the fungal community in tolerant ashes. Additional OTUs with higher amounts in tolerant plants (e.g., OTU0014, 0026, and 0036) belonged to *Microstroma*, uncl. *Dothidiomycetes* and *Neosetophoma*. However, the abundances of these taxa were comparably low.

An indicator species analysis at the OTU level revealed four groups for the tolerant ash trees. It again concerns *P. flavescens* (OTU0003), but *Microstroma album* (OTU0014), *Vishniacozyma dimennae* (OTU0139) and *Exobasidium* spp. (OTU0141) could also be proven (*p* < 0.05).

#### Culturable Fungal Communities

In addition to the marker gene analysis, the culturable leaf-associated fungal community was compared between tolerant and susceptible ash trees. The population densities of the fungi ranged between 1 × 10^4^ and 4 × 10^5^ CFU/g fresh weight with no significant differences depending on the health status. Previous studies showed that the leaf fungal density is very changeable with yeasts ranging between 10 and 10^10^ and filamentous fungi between 10^2^ and 10^8^ CFU/g (Thompson et al., 1993; Inacio et al., 2002). In total, 931 filamentous isolates were classified *via* ITS rRNA sequencing and 773 yeast-like isolates *via* MALDI-TOF MS. The 1,704 isolates were grouped at the species level into 49 phylotypes belonging to 42 genera.

The majority of fungal isolates was assigned to the phylum *Ascomycota* (72%) followed by *Basidiomycota* with 28%. At the class level, *Dothideomycetes* (63%), and *Tremellomycetes* (28%) dominated the community. At the species level, a clear dominance of the saprophytic yeasts *A. pullulans* (50.5%), *P. flavescens* (14.1%), and *V. carnescens* (9%) was observed. The most abundant species *A. pullulans*, which is known for its switch between yeast-like and filamentous growth mode, accounted for 28% of the yeasts and 69% of the filamentous fungi. Thus, the filamentous fungi were clearly dominated by *A. pullulans*, whereas *P. flavescens* was the predominant yeast (31%).

**FIGURE 4:**
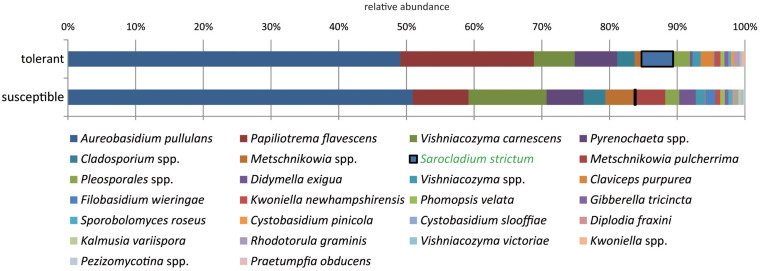
Comparison of the culturable fungal community from tolerant and susceptible ashes at the species level. The species significantly increased in resistant trees is highlighted.

When comparing trees with different health statuses, *P. flavescens* occurred in higher abundance in tolerant trees (Figure 4). However, this difference could not be statistically ensured. *Sarocladium strictum* isolates made up a lower proportion but showed a significant difference with a 24-fold increase in tolerant trees.

### *In vitro* Antagonistic Activity of Fungal Isolates

#### Yeast-Like Fungi

To detect potential antagonists, the yeast isolates were screened for growth inhibition of *H. fraxineus* P3. Overall, the screening revealed a high proportion of isolates with slight inhibitory effects. One-fifth of the isolates inhibited *H. fraxineus* by 31–50%. The potential antagonists were obtained in a comparable ratio from susceptible and tolerant trees. They were dominated by *A. pullulans* (56%), followed by the *Basidiomycota* members *P. flavescens* (23%) and *Vishniacozyma* spp. (13%). Other taxa such as *Metschnikowia* sp., *Cystobasidium pinicola* and *Kwoniella newhampshirensis* were represented in frequencies between 5% and less than 1%. The highest inhibition rates were achieved by *A. pullulans* isolates with 37% on average, while the mean inhibition of the other taxa was slightly lower with 32–34%.

**FIGURE 5:**
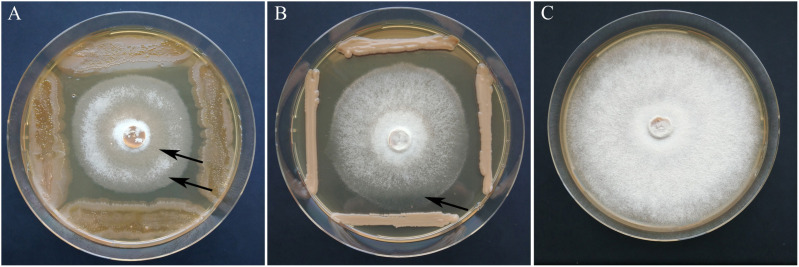
Growth inhibition of *H. fraxineus* P3 after cocultivation with *A. pullulans* A3K040 **(A)** and *P. flavescens* A3P071 **(B)** in comparison to the control **(C)**. The clearing of the *H. fraxineus* mycelium which indicates an ongoing lysis of the pathogen is marked by arrows.

Based on the primary screening, 28 isolates with an inhibition rate above 30% were subjected to the statistical test on antagonism against two *H. fraxineus* isolates. The isolates were derived from all the locations and from both susceptible and tolerant trees. An overview on the antagonistic potential of the yeasts is given in Table 1. In total, six of the 28 isolates significantly inhibited the growth of *H. fraxineus* HF23 at 39–50%. The *H. fraxineus* strain P3 was inhibited by these yeasts over a similar range, but this finding could not be statistically ensured. After cocultivation, the vitality of the remaining *H. fraxineus* mycelium was also examined. Ten out of the 28 isolates were able to completely kill *H. fraxineus* P3, whereas the vitality of HF23 was only slightly injured. Remarkably, four of the nine isolates of *P. flavescens*, a species that was found to have increased in tolerant trees, were able to devitalize *H. fraxineus* P3. When summarizing all the antagonistic features, the best isolates were A3P046, A3K040, and A3P071. D3K042 and C4K010 showed a strong fungistatic effect during the cocultivation test indicated by the ability of the pathogen to regrow. The antagonistic activity is exemplarily shown for isolates A3K040 and A3P071 in Figure 5. In addition to the growth inhibition, a lightening of the mycelium was visible, indicating an ongoing lysis that started approximately 10 days after cocultivation. This effect was differently pronounced in both isolates.

**TABLE 1:** Antagonistic activity of yeast-like epi/endophytic fungi as assessed by the growth inhibition of *H. fraxineus* and the vitality of the remaining pathogen mycelium.

**Isolate**	**Taxonomic assignment**	**Growth inhibition rate in**		**Vitality of mycelium (% of**	
		**dual culture (%)^a^**		**untreated control)**	
		**P3**	**HF23**	**P3**	**HF23**
A3K052	*Aureobasidium pullulans*	39.2	37.3	54.9	92.2
A3P046	*Aureobasidium pullulans*	41.0	45.7*	0.0	89.3
A4K061	*Aureobasidium pullulans*	41.0	35.8	61.1	84.0
A2P099	*Aureobasidium pullulans*	40.7	36.7	0.0	85.5
C1P011	*Aureobasidium pullulans*	32.5	37.5	0.0	85.3
A3K040	*Aureobasidium pullulans*	34.6	39.2*	0.0	89.5
A1K043	*Aureobasidium pullulans*	41.3	39.8*	71.8	88.8
C4K081	*Aureobasidium pullulans*	37.5	40.0*	35.7	68.1
D1P037	*Aureobasidium pullulans*	44.9	37.8	100.0	87.0
B4P060	*Aureobasidium pullulans*	38.3	37.0	95.3	76.8
D3P009b	*Cystobasidium pinicola*	16.7	32.9	102.0	115.4
B3K004	*Metschnikowia pulcherrima*	37.3	34.1	87.0	84.9
C3P008	*Metschnikowia* sp.	25.9	35.8	0.0^b^	73.6
A3P071	*Papiliotrema flavescens*	25.9	38.3	0.0	93.3
A4P066	*Papiliotrema flavescens*	23.5	30.3	0.0	87.8
B3P048	*Papiliotrema flavescens*	27.2	25.3	0.0	100.0
A2P025	*Papiliotrema flavescens*	25.6	35.0	0.0	91.9
C2P072	*Papiliotrema flavescens*	23.5	33.3	100.0	66.7
C2K025	*Papiliotrema flavescens*	25.9	35.8	100.0	87.5
D4K115	*Papiliotrema flavescens*	25.0	37.8	94.0	97.2
C4K058	*Papiliotrema flavescens*	25.7	31.3	92.3	76.0
C1P081	*Papiliotrema flavescens*	25.9	37.8	81.9	94.5
D3K042	*Papiliotrema flavescens*	33.3	50.0*	93.8	82.1
A3P017	*Vishniacozyma carnescens*	27.5	32.1	85.3	75.0
C2K045	*Vishniacozyma carnescens*	25.9	29.6	96.9	104.3
C4K010	*Vishniacozyma carnescens*	30.8	47.5*	79.6	76.9
C4K012	*Vishniacozyma* sp.	28.6	35.4	0.0^b^	98.6
B3P084	*Vishniacozyma* sp.	32.1	29.3	58.7	89.7

^*a*^Growth inhibition was tested on the *H. fraxineus* isolates P3 and HF23. *Significantly increased in comparison to the control *M. pulcherrima* (B3K065), which did not show antagonistic activity in dual cultures (growth inhibition rates: 28.4 and 15.8). The test was performed by Kruskal-Wallis one-way analysis of variance on ranks followed by pairwise comparisons with Dunn’s method (*P* < 0.05). ^*b*^Detection of the antagonistic isolate inside the recovered *H. fraxineus* mycelium or instead of the mycelium.

#### Filamentous Fungi

Due to the high proportion of *A. pullulans* among the filamentous fungi, more than half of the fungi tested here belonged to this species. To avoid testing clones of the same strain, only a selection of *A. pullulans* strains was tested per sample. Accordingly, the screening of filamentous fungi included 695 isolates. As a result, 79 isolates (11.4% of the total isolates) inhibited the pathogen growth by at least 43%, i.e., higher than the mean self-inhibition rate of *H. fraxineus* P3. The highest values were determined for the *Cladosporium* isolates C1K002 and A3K053 with inhibition rates of 70 and 60%. The positively screened isolates obtained in comparable proportions from tolerant and susceptible trees represented members of *Aureobasidium* (40%), *Pyrenochaeta* (16%), *Cladosporium* (10%), *Gibberella* (10%), and *Pleosporales* (8%) as well as *Diplodia, Didymella, Sarocladium*, and *Phomopsis* with lower occurrence. The inhibition rates per group varied between 41 and 55%.

Most likely due to the different test procedures, the filamentous isolates inhibited *H. fraxineus* P3 by 48%, whereas the mean inhibition rate of yeast-like isolates was 37%. Otherwise, not only the procedure but also the morphological status of the fungal isolate could be of importance for the antagonistic properties of fungi.

Out of the 79 antagonistic isolates, 37 were derived from different samples and taxonomic groups. To exclude clones from the subsequent statistical test, only these isolates were selected. As shown in Table 2, four isolates suppressed either *H. fraxineus* strain significantly with inhibition rates from 72 to 100%. The inhibitory effects were distinctly above the level of self-inhibition for *H. fraxineus*. Five further isolates inhibited one of the *H. fraxineus* strains significantly with rates between 57 and 93%.

**TABLE 2:** Antagonistic activity of filamentous epi/endophytic fungi as assessed by the growth inhibition of *H. fraxineus* and a vitality test of the remaining pathogen mycelium.

**Isolate**	**Taxonomic assignment**	**Growth inhibition rate in**		**Vitality of mycelium (% of**	
		**dual culture (%)^a^**		**untreated control)^a^**	
		**P3**	**HF23**	**P3**	**HF23**
A1P017	*Aureobasidium pullulans*	43.8	42.5	90.8	42.3^b^
A3K056	*Aureobasidium pullulans*	43.8	48.3	90.8	80.8^b^
A1K012	*Aureobasidium pullulans*	43.8	41.4	89.5	70.5^b^
A3P027	*Aureobasidium pullulans*	41.9	42.5	90.8	102.6
A4P023	*Aureobasidium pullulans*	44.8	52.9	76.3^b^	93.8^b^
B1K008	*Aureobasidium pullulans*	42.9	52.9	76.3	102.6
B3P001	*Aureobasidium pullulans*	44.8	46.0	90.8	32.1^b^
B4P025	*Aureobasidium pullulans*	45.7	42.5	88.2	56.3
C1P010	*Aureobasidium pullulans*	43.8	20.9	90.0	94.8
C3K025	*Aureobasidium pullulans*	41.9	34.8	90.0	3.4^b^
C1K002	*Cladosporium* sp.	100.0*	100.0*	0.0*	0.0
D1K008	*Cladosporium* sp.	87.2*	100.0*	20.0^b^	0.0
A3K053	*Cladosporium* sp.	75.8*	71.6*	36.8^b^	0.0^b^
D1K021	*Cladosporium* sp.	42.8	74.7	60.0^b^	0.0^b^
C2K082	*Cladosporium* sp.	44.8	93.3*	86.0^b^	0.0
B4K021	*Didymella exigua*	44.8	42.8	90.0^b^	86.2^b^
D1K016	*Diplodia fraxini*	49.7	59.6	0.0*^,b^	8.6^b^
C4P019	*Gibberella tricincta*	72.1*	71.2	80.0	86.2^b^
C4K037	*Gibberella tricincta*	75.6*	86.4*	76.0^b^	95.0
B2K028	*Phomopsis velata*	55.6	52.9	0.0*^,b^	0.0^b^
B4K006a	*Phomopsis velata*	36.9	27.6	92.0^b^	94.8^b^
B4K006b	*Phomopsis velata*	46.7	39.4	50.0^b^	17.2^b^
B1P004	*Phomopsis velata*	51.7	16.5	0.0*^,b^	85.7^b^
B2K017	*Pleosporales* spp.	59.6	57.8	86.6	100.0
B2K022	*Pleosporales* spp.	66.7*	54.9	81.6^b^	100.0
B1P005	*Pleosporales* spp.	45.4	54.5	86.0	89.3
C2K088	*Pleosporales* spp.	22.4	−3.8	88.0	76.9
A4K021	*Pyrenochaeta* spp.	44.7	26.6	78.3	100.0
A4P009	*Pyrenochaeta* spp.	67.5*	31.4	80.3	95.0
B1K010	*Pyrenochaeta* spp.	57.5*	51.0	78.9	95.0
A1K007	*Pyrenochaeta spp.*	52.1	28.1	80.6	91.3
B4P008	*Pyrenochaeta spp.*	51.1	26.6	81.2	87.5
B3P007	*Pyrenochaeta spp.*	50.0	28.1	83.6	100.0
C3P017	*Pyrenochaeta spp.*	40.8	7.6	88.0	83.9
C2K078	*Pyrenochaeta spp.*	38.2	7.6	88.0	90.3
D3P026-P	*Sarocladium strictum*	46.2	40.0	55.1	107.7
C2P015	*Sarocladium strictum*	33.7	42.4	80.0	53.6^b^
Self-inhibition	*H. fraxineus*	49.3	43.6	92.4	97.2

^*a*^Growth inhibition was tested on the *H. fraxineus* isolates P3 and HF23. *Significantly increased in comparison to control *Didymella unclass.* (B4K020), which did not show antagonistic activity in dual cultures (growth inhibition rates: 20.4 and 19.8). The test was performed by Kruskal-Wallis one-way analysis of variance on ranks followed by pairwise comparisons with Dunn’s method (*P* < 0.05). ^*b*^Detection of the antagonistic isolate inside the re-cultivated *H. fraxineus* mycelium.

**FIGURE 6:**
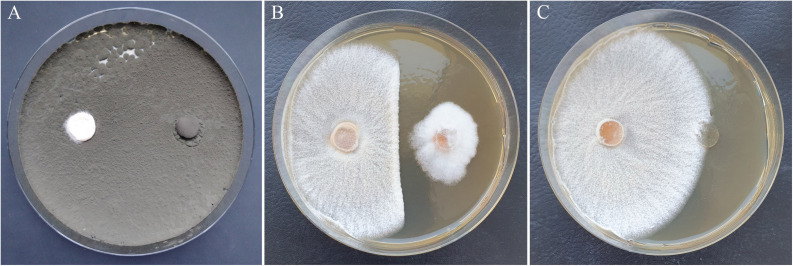
Growth inhibition of *H. fraxineus* HF23 after cocultivation with *Cladosporium* sp. C1K002 **(A)** and *Sarocladium strictum* D3P026-P **(B)** in comparison to the *H. fraxineus* control **(C)**. Left plate side: *H. fraxineus*, right plate side: agar plug with antagonist **(A, B)** or without mycelium **(C)**.

The most effective isolates C1K002 and D1K008, which inhibited the pathogen almost completely, were members of the genus *Cladosporium*. Both isolates showed a rapid growth by strong sporulation and were already able to suppress the *H. fraxineus* strains during the first week of cocultivation. Accordingly, the growth of *H. fraxineus* was affected even in its initial phase. After 14 days, no or very few mycelia from *H. fraxineus* had grown (Figure 6 and Table 2). A third *Cladosporium* isolate (A3K053) showed an almost comparable antagonistic activity, with inhibition rates of 72 to 76%. Additionally, B2K028 and D1K016 were able to kill the pathogen more or less completely. This killing was achieved by the ingrowing of the antagonistic fungus into the *H. fraxineus* colony. Consequently, the antagonistic isolate was predominantly detected in the re-cultivation approach. The fourth isolate with clear significant effects, C4K037, was identified as *Gibberella tricincta.* Like other strains of this species, this isolate also tended to invade the *H. fraxineus* colony, which partly led to its occurrence in the re-cultivated mycelium of the pathogen (Table 2). In general, fungicidal effects that became visible during the re-cultivation approach have been observed within several taxonomic groups. These effects particularly concern isolates of fast-growing species: for example, *Diplodia fraxini*, *Cladosporium* sp., *Phomopsis velata*, and to a lesser extent, *A. pullulans*, and *S. strictum.*

## Discussion

To identify fungal taxa associated with tolerance to *H. fraxineus*, we analyzed the fungal communities of ash leaves by ITS2 amplicon sequencing and culturing. Leaf-colonizing fungi are considered as a barrier to invaders such as *H. fraxineus* that spread starting from leaf infections (Cross et al., 2017; Hanackova et al., 2017b). The indigenous fungi fight against the invading pathogen directly by resource and space competition, partly supported by antibiosis, parasitism or indirectly by the induction of plant resistance (Busby et al., 2016; Jia et al., 2020).

Our study was based on the assumption that both epi- and endophytic fungi interact with the pathogen in a similar way. During and directly after the invasion and penetration of epidermal tissue, the pathogenic fungus is in contact with both habitats, the phyllosphere and endosphere of the ash leaves. Aside from that, several indigenous microorganisms can switch between the two habitats (Hardoim et al., 2015; van Overbeek and Saikkonen, 2016). Accordingly, both habitats were sampled in a combined approach.

Culture-independent as well as culturing approaches showed a strong presence of saprophytic yeasts, especially within the phyllosphere communities (Slavikova et al., 2007; Davydenko et al., 2013; Hanackova et al., 2017a; Kemler et al., 2017). Yeasts thrive on plant exudates, whereas plants can benefit from the secondary metabolites produced by yeast, e.g., auxins, chelates, and glycolipids (Fonseca and Inacio, 2006; Kemler et al., 2017). In addition, several yeasts possess antifungal activities based on resource competition, antibiosis, biofilm formation or the production of volatile organic compounds (Liu et al., 2013; Freimoser et al., 2019). Therefore, an intensive colonization of leaves with yeasts can contribute to higher plant fitness and pathogen resistance. While yeasts are very common in the phyllosphere, the endophytic community is dominated by filamentous fungi (Prior et al., 2017). In our study, the fungal community was clearly dominated by the yeast-like fungus *A. pullulans*. This species is a very abundant colonizer of plant surfaces but was also often isolated as an endophyte from trees (Albrectsen et al., 2010; Martin-Garcia et al., 2012), including common ash (Slavikova et al., 2007; Davydenko et al., 2013; Hanackova et al., 2017a). As a pleiomorphic fungus, it is known to switch between yeast and mycelial forms in response to its environmental conditions (Ramos and García Acha, 1975; Campbell et al., 2004). Among the next-most abundant fungi, *P. flavescens* and *V. carnescens* belong to yeasts, while *Pyrenochaeta* spp. and *Cladosporium* spp. have filamentous growth habits.

The fungal communities of tolerant and susceptible *F. excelsior* trees showed a similar genetic diversity and community structure. Nevertheless, the differential abundance analysis and the indicator species analysis revealed several OTUs as well as an isolate group with significant relations to the health status of the trees. In addition to the predominant *P. flavescens* OTU0003, several less abundant OTUs and the isolate group *S. strictum* could be detected. Strains from the genus *Papiliotrema* are known for their protective effect against fungal disease-related pre- and postharvest losses of different crops. Especially isolates of *P. flavescens* (formerly *Cryptococcus flavescens*) are widely used as biocontrol agents against head blight on cereals caused by *Fusarium graminearum* (Dunlap and Schisler, 2010; McSpadden Gardener et al., 2014; Schisler et al., 2014). Similarly, the postharvest infestation of different fruits with common pathogens such as *Botrytis cinerea*, *Alternaria alternata*, and *Penicillium expansum* were shown to be reduced effectively by *P. flavescens* and *P. laurentii* strains (Qin and Tian, 2004; Zhang et al., 2007; Kheireddine et al., 2018). Various *Sarocladium* strains are discussed as potential antagonists due to the formation of antifungal substances such as antibiotics and hydrolytic enzymes (Wicklow et al., 2005; Guimarães et al., 2017). In addition, *S. strictum* has been shown to have disease-reducing effects through mycoparasitism, e.g., on *Botrytis cinerea* and *Helminthosporium solani* (Rivera et al., 2007; Choi et al., 2008). However, the use of *S. strictum* as a biocontrol agent might be limited due to the putative human pathogenic potential of this species (Schell and Perfect, 1996; Sharma et al., 2013). Consequently, the comprehensive analysis by applying a marker gene study and a culturing approach resulted in two distinct fungal groups which are specific to tolerant ash trees and might be associated with the ability of the trees to resist the pathogen.

As a result of the cocultivation test on antagonism, several yeast isolates were found to possess an antagonistic capacity by a direct growth reduction of *H. fraxineus* (of up to 50%) and/or an ongoing mycelium lysis, resulting in the complete killing of one of the pathogen strains. Cell-lytic effects from the secretion of exoenzymes have been described for several antagonistic yeasts (Bar-Shimon et al., 2004; Zhang et al., 2011, 2012). The lysis of fungal cell walls usually involves various synergistically acting enzymes, including glucanases, chitinases and proteases (Salazar and Asenjo, 2007). In particular, numerous strains of *A*. *pullulans* harbor a broad spectrum of lytic enzymes. This enzymatic activity as well as the production of other antimicrobial compounds make *A. pullulans* a versatile biocontrol agent (Federici, 1982; Prasongsuk et al., 2018). Similar to *P. flavescens*, *A*. *pullulans* but also *Metschnikowia* and *Vishniacozyma* strains can effectively mitigate pre- and postharvest infections (Mari et al., 2012; Gramisci et al., 2018; Pawlikowska et al., 2019).

Compared to the yeasts, the filamentous fungi usually showed stronger antagonistic effects, even if the differences of the applied tests were taken into account. Three *Cladosporium isolates* (C1K002, D1K008, and A3K053) suppressed both *H. fraxineus* strains significantly with inhibition rates of 72–100%. The strong effects were also indicated by a clear reduction in the pathogen vitality. Fungicidal effects were also observed for *Diplodia fraxini* D1K016 and *Phomopsis velata* B2K028. All these isolates were representatives of fast-growing species that affected and displaced the pathogen in dual culture by strong sporulation or by overgrowing. Hanackova et al. (2017a) tested a number of endophytic fungi of common ashes in a similar approach and found comparable high inhibition rates. The best results were also achieved for fast-growing species, e.g., *Botrytis cinerea* and *Phoma macrostoma*. However, *Diplodia fraxini*, species of *Phomopsis* (syn. *Diaporthe*) and *Botrytis cinerea* are also known as plant pathogens (Elena et al., 2018; Hua et al., 2018; Sessa et al., 2018). Moreover, a promising antagonist (*Hypoxylon rubiginosum*) was also found through the detection of the antifungal metabolite phomopsidin, although the *in vitro* inhibitory effect was less pronounced (Halecker et al., 2020). Remarkably, studying endophytic fungi from dieback-tolerant ash species including *F. mandshurica* and *F. ornus* resulted in somewhat lower antagonistic activity against *H. fraxineus*, with inhibition rates of 40–64% (Kosawang et al., 2018).

Three of the isolates with the highest antagonistic activity were members of the genus *Cladosporium.* This genus was found to be the second-most abundant taxon among the leaf fungal communities of the dieback-tolerant Manchurian ash (Cleary et al., 2016). Furthermore, *Cladosporium* is closely related to the genus *Mycosphaerella* (Braun et al., 2003), which represented the predominant fungal group of the Manchurian ash (Cleary et al., 2016). Both genera include numerous widespread species varying in their ecological roles, including plant pathogens, mutualistic endophytes, saprophytes or hyperparasites on other fungi (Crous, 2009; Bensch et al., 2012). The ability of *Cladosporium* species to parasitize other fungi, in particular those causing rust diseases of agricultural and forestry crops (i.e., *Puccinia* sp.) has been demonstrated for decades in numerous studies (Tsuneda and Hiratsuka, 1979; Moricca et al., 2005; Zhan et al., 2014; Torres et al., 2017). One *Cladosporium* species (*C. delicatulum*) was also found to be a mycoparasite on *Taphrina pruni*, the plum gall pathogen of *Prunus* species (Amanelah Baharvandi and Zafari, 2015). Thus, recent data indicate that *Cladosporium* species have potential in plant diseases management. However, further studies are needed to clarify the biology of the antagonism. Since some *Cladosporium* strains can also be pathogenic to vertebrate hosts, including humans (Sandoval-Denis et al., 2016), the biosafety of the respective strains must be guaranteed on a case-specific basis (Torres et al., 2017).

The two *H. fraxineus* strains used in the dual culture experiment showed a different sensitivity to a range of antagonists. The *H. fraxineus* strain P3 was more damaged by some yeast isolates, while HF23 showed a higher sensitivity to individual filamentous fungi, including isolates of *Cladosporium*. *H. fraxineus* is known for its intrinsic diversity, which was indicated by the differing growth rates, enzymatic activity and virulence of various isolates (Junker, 2013; Junker et al., 2017). Against the background that the ash dieback invasion of Europe started with only two strains, a further increase in genetic diversity can be expected (McMullan et al., 2018). Accordingly, subsequent *in planta* tests of antagonistic activity should be performed with infected petioles representing the natural *H. fraxineus* populations from a typical ash forest. It remains unclear to what extent our results from the *in vitro* studies can be confirmed using this approach. Another critical aspect of *in vitro* testing is the lack of interactions of the antagonists with the plant holobiont. The complex plant-microbiota interaction can lead to both a reduction and an enhancement of the desired effect (Knudsen et al., 1997; Schulz et al., 2015; Latz et al., 2018).

In conclusion, the comparative analysis of the leaf-inhabiting fungi from susceptible and tolerant ashes revealed a high similarity in the community structure. Only a few fungal groups primarily belonging to *P. flavescens* and *S. strictum*, showed a distinctly higher abundance in tolerant ashes. These specific groups are suggested to improve pathogen tolerance, which could be mediated by competition, by inducing a systemic resistance or just by altering the microbial community structure (Pieterse et al., 2014; Romera et al., 2019). Additionally, *in vitro* tests of antagonism against *H. fraxineus* indicated clear inhibitory effects of various yeasts and particularly of several fast growing filamentous fungi. Both, the yeasts with the relatively mild inhibitory effects and the filamentous species with stronger activities, particularly *Cladosporium* sp. C1K002 and D1K008, could be involved in disease suppression. Based on these results, for the next step, *in planta* tests are necessary to evaluate these promising groups and isolates with respect to their efficacy and stability in inhibiting *H. fraxineus*. In this context, possible shifts in the microbial community should be studied and might be correlated with the plant health as well.

## Data Availability Statement

The datasets presented in this study can be found in online repositories. The names of the repository/repositories and accession number(s) can be found below: https://www.ncbi.nlm.nih.gov/genbank/, PRJNA611938.

## Author Contributions

RB and KU performed the experiments, analyzed the data, and prepared the manuscript. UB performed the MALDI-TOF MS analysis. KU, MK, and AU conducted the microbiome analysis. All authors listed here substantially contributed to and approved the manuscript. AU and MK supervised the entire study.

## Conflict of Interest

The authors declare that the research was conducted in the absence of any commercial or financial relationships that could be construed as a potential conflict of interest.
